# Thiamine (Vitamin B1)—An Essential Health Regulator

**DOI:** 10.3390/nu17132206

**Published:** 2025-07-02

**Authors:** Julia Kaźmierczak-Barańska, Krzysztof Halczuk, Bolesław T. Karwowski

**Affiliations:** DNA Damage Laboratory, Food Science Department, Faculty of Pharmacy, Medical University of Lodz, ul. Muszynskiego 1, 90-151 Lodz, Poland; krzysztof.halczuk@umed.lodz.pl (K.H.); boleslaw.karwowski@umed.lodz.pl (B.T.K.)

**Keywords:** thiamine, antioxidant, oxidative stress, mitochondria/ROS

## Abstract

Thiamine (vitamin B1) is key in maintaining cellular health and energy metabolism. Thiamine is required for proper functioning of enzymes involved in glucose metabolism, which is critical for providing energy to cells. This energy is essential for various cellular processes, including DNA repair mechanisms. In addition, it is a prerequisite for the functioning of key enzymes in the biosynthesis of pentose sugars, which are essential in the synthesis of nucleic acids. Additionally, thiamine has antioxidant properties that help reduce oxidative stress in cells; thus, by relieving this stress, thiamine indirectly supports the maintenance of DNA integrity. Ensuring adequate thiamine intake through diet or supplements can support overall cellular health and potentially aid in DNA repair processes. This review aims to highlight the essential role of vitamin B1 in supporting metabolic health, especially given that deficiencies can develop in patients with disease-related malnutrition as well as in those with an inadequate diet.

## 1. Introduction

Thiamine is a critical and rate-limiting cofactor for many enzymes, including those acting at entry points and critical junctions for glucose, fatty acid, and amino acid pathways. It is characterized by a short half-life and limited storage capacity. Thiamine is susceptible to degradation and depletion by various products, such as environmental and pharmaceutical chemicals or food additives that characterize modern life. Thiamine deficiency is primarily associated with alcoholism; however, in the absence of a medical history of alcoholism, the variable symptoms of thiamine deficiency (multi-organ effects) can be attributed to other conditions and easily misdiagnosed. Moreover, studies show a wide range of probable causes of thiamine deficiency, including disease-related malnutrition (usually combined with significant, unintentional weight loss), gastrointestinal problems, recurrent vomiting, surgical complications, food insecurity, chronic use of diuretics (for treating heart failure), and adherence to monotonous or restrictive diets [1]. The purpose of this review is to highlight the vital role of vitamin B1 in maintaining metabolic health, particularly given that deficiency can arise in individuals with poor dietary habits or impaired absorption.

## 2. Effects of Inadequate Intake

The established daily requirement for thiamine in adults is 1.1–1.2 mg, a figure that has been consistent for decades (Table 1) [2]. Given this low recommended dietary allowance (RDA) for thiamine and the widespread consumption of fortified foods, one might assume that thiamine deficiency (TD) is rare in developed countries with food security. However, it may still occur in certain populations or medical situations, such as patients with HIV/AIDS or pregnant women experiencing prolonged hyperemesis gravidarum [3]. Due to this fact, thiamine levels are not consistently assessed in healthcare practices or nutritional studies [4,5]. Reports indicate that thiamine content in in thiamine-fortified foods is sometimes four times higher than the recommended daily allowance (RDA). However, thiamine deficiency is frequently observed. The problem is under-recognized rather than limited to traditionally defined populations [1,6,7,8]. A study by Kim et al. [9] suggests that consuming the RDA of thiamine (1.29 mg/day) may not be enough to achieve optimal thiamine status. Early symptoms of thiamine deficiency are nonspecific and can easily be attributed to various other health issues. Common symptoms include persistent or unusual fatigue, mood swings, excessive irritability, mood lability, feelings of brain fog, subtle memory impairment, loss of appetite, sleep disturbances, and gastrointestinal discomfort or dysmotility [10]. Over the past few decades, urbanization, industrialization, economic development, and globalization have drastically transformed lifestyles and dietary patterns, leading to an increased intake of highly processed foods. This shift often involves an excessive supply of sodium, saturated fatty acids, and free sugars. Additionally, increasing carbohydrate intake can decrease plasma and urine thiamine concentrations without affecting enzyme activity. Therefore, relying heavily on fortified carbohydrate sources in our diets can result in an inadequate supply of thiamine [11]. A large cross-sectional study conducted between 2014 and 2018 and involving 34,700 individuals, indicated a downward trend in thiamine intake levels [12]. Various parameters were assessed, and health questionnaires were used to classify demographic status and healthy lifestyle. The study found an association between dietary thiamine intake and prevalence of cardiovascular diseases, diabetes, and mental health issues. The results suggest that adequate thiamine intake may reduce the risk of type 2 diabetes, cardiovascular diseases such as hypertension, heart attack, or angina, and also be a factor for mental health. Studies involving high doses of vitamin B1 (Table 1) suggest that thiamine supplementation (300 mg/day) may have a beneficial effect on blood pressure in individuals with hyperglycemia and could play a role in preventing further vascular complications [13]. In addition, thiamine at this dosage may enhance glucose tolerance in individuals with early-stage hyperglycemia and potentially help prevent or delay the onset of type 2 diabetes in those with impaired glucose regulation [14]. Furthermore, high-dose thiamine (400 mg/day) may be considered as a support for therapy in the case of stroke. In an animal model of stroke induced by carotid artery occlusion, thiamine was shown to reduce glutamate levels, which is a major factor in excitotoxicity and neuronal damage [15]. As other studies have indicated, low serum thiamine levels are associated with a high prevalence of depressive symptoms [16,17].

## 3. Energy Metabolism and Cellular Function

**Figure 1:**
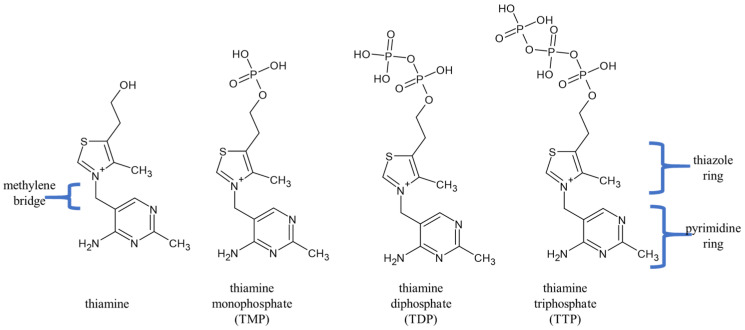
Thiamine phosphorylated forms.

Thiamine, also known as vitamin B1, is a water-soluble vitamin that consists of a pyrimidine ring connected by a methylene bridge to a thiazole ring. In human tissues, thiamine [19] mainly exists in phosphorylated forms (Figure 1), including thiamine monophosphate (TMP); thiamine diphosphate (TDP) [20], also known as thiamine pyrophosphate); and thiamine triphosphate (TTP) [21], which also exists in a non-phosphorylated form (thiamine). TDP serves as a coenzyme for over 20 known enzymes involved in various metabolic pathways (e.g., transketolases, 2-Hydroxyacyl-CoAlase, pyruvate dehydrogenase, α-ketoglutarate dehydrogenase complex) [3]. These enzymes play a role in catabolic processes that lead to ATP synthesis, NADP reduction, and the biosynthesis of pentoses, which in turn are essential for nucleic acid synthesis, ensuring its stability and integrity.

Thiamine in food is mainly found in phosphorylated forms in animal products, whereas the non-phosphorylated form predominates in plant products. After ingestion, intestinal phosphatases hydrolyze thiamine phosphate esters. Thiamine is absorbed through the mucosal membrane [18]. In healthy individuals, thiamine absorption is above 95% with adequate intake. Thiamine in the blood is mainly found in erythrocytes (>80% of total blood thiamine) in the form of thiamine diphosphate and TTP, while low amounts of the vitamin are present in plasma as free thiamine, TMP, and protein-bound TDP [2]. In the human body, thiamine concentrations are generally highest in the skeletal muscle, heart, brain, liver, and kidneys [3].

Thiamine is phosphorylated in tissues by thiamine diphosphokinase, which catalyzes the formation of thiamine diphosphate using ATP [22]. In mitochondria, thiamine diphosphate acts as a cofactor for enzymes in the Krebs cycle, playing a key role in processes related to mitochondrial energy metabolism. The Krebs cycle, also known as the citric acid cycle or tricarboxylic acid cycle, is one of the stages of aerobic cellular respiration. Its products are used to synthesize a large number of ATP molecules during oxidative phosphorylation, where thiamine diphosphate plays an important role in the oxidative decarboxylation of pyruvic acid and α-ketoglutaric acid. Additionally, thiamine diphosphate acts as a coenzyme for transketolase (TKT) in the pentose phosphate pathway (PPP), which is an important alternative to the glycolysis–Krebs cycle pathway. In the pentose pathway, glucose is converted into ribose, being an essential component for DNA and RNA synthesis, with the production of reduced forms of nicotinamide adenine dinucleotide phosphate (NADPH), which is necessary for steroid hydroxylation, fatty acid synthesis (myelin), and antioxidant enzymes during oxidative stress (glutathione and thioredoxin) [2,23,24,25]. Both ribose and NADPH are essential for proper cell function. Transketolase plays a critical role in this process, and its activity relies on binding with thiamine diphosphate. Transketolase activity decreases under thiamine deficiency conditions, while no accumulation of the apoenzyme is observed; instead, downregulation of mRNA occurs. This observation suggests that thiamine may regulate the expression of genes encoding enzymes that require thiamine diphosphate as a cofactor [26,27].

The PPP is an alternative route of glucose metabolism, accounting for no more than 10% of glucose utilization [28]. It comprises two phases: an irreversible oxidative phase and a reversible non-oxidative phase. Its primary function is to supply the cell with the reducing agent NADPH, which maintains intracellular GSH levels, and ribose-5-phosphate, which can be used in the de novo synthesis of nucleic acids. For these reasons, cells with the active PPP are protected against oxidative stress and xenobiotics-generating ROS and have a theoretical proliferative advantage. This pathway is most active where the demand for NADPH is high (in the liver, adipose tissue, erythrocytes) [29]. Glucose-6-phosphate dehydrogenase (G6PD) is an enzyme of the PPP that catalyzes the oxidation of glucose-6-phosphate to 6-phosphoglucono-δ-lactone, with simultaneous reduction of NADP+ to NADPH [30]. This is the most important regulatory step of this pathway. The PPP meticulously modulates its activity to meet divergent metabolic requirements of different tissues. In rapidly proliferating tissues (bone marrow and some neoplastic tissues), the PPP directs its metabolites toward nucleotide synthesis, providing necessary precursors for DNA and RNA synthesis, thus meeting the requirements of cell proliferation. Conversely, in tissues with high biosynthetic activity, particularly fatty acid and sterol synthesis, NADPH produced by PPP supports antioxidant mechanisms. The multidirectionality of the PPP demonstrates how cells masterfully utilize metabolic pathways, adapting them to current physiological, biosynthetic, and defense needs, thereby maintaining a harmonious cellular and systemic physiological state. Both phases of the PPP play a crucial role in orchestrating metabolic pathways to meet the demands of cell growth and maintenance. In human fibroblasts, ATM kinase (mutated in ataxia-telangiectasia), which is activated in response to DNA double-strand breaks, induces G6PD activity, increases glucose flux through the PPP, reduces ROS levels, increases deoxyribonucleotide synthesis, and enhances the synthesis of deoxyribonucleotides, facilitating effective DNA repair. However, when G6PD is silenced, the ability to repair DNA double-strand breaks is significantly compromised [31]. In ataxia, mutated ATM does not interact with the PPP pathway, resulting in reduced antioxidant potential (low NADPH) and inhibition of DNA repair due to the lack of substrates for DNA synthesis. Other studies also indicate increased susceptibility to DNA damage and UV-induced apoptosis in G6PD-deficient patients. Reduced glutathione levels are significantly lower in mononuclear cells with G6PD deficiency than in healthy controls, and more DNA damage has been observed [32]. Increased G6PD activity, which provides more reducing equivalents, has an anti-apoptotic and pro-survival role. Active PPP not only ensures better survival and escape from apoptosis but also becomes a pro-carcinogenic factor and enables effective DNA repair [33,34]. Interestingly, in this context, p53 (the guardian of the genome) negatively modulates PPP activity [35]. The loss of functional p53, which occurs in many cancer cells, can unleash the PPP, promote tumor survival, and make cells more resistant to oxidative stress. Most normal cells exhibit transketolase activity and undetectable or very low activity of transketolase-like proteins (TKTL enzymes). In contrast, TKTLs are present in malignant tissues and are considered proto-oncogenes, utilizing thiamine as a cofactor [36]. In response to DNA damage caused by radiation, the protein p53 binds to DNA, inhibiting replication and cell proliferation and also possibly triggering apoptosis. Thiamine and thiamine diphosphate have been shown to affect p53-DNA binding and p53-dependent functions, such as replication and the apoptosis induced by ionizing radiation [37]. Thiamine inhibited p53-DNA binding, induced by ionizing radiation, effectively abolishes the antiproliferative and antireplicative activity of p53. Additionally, in irradiated cells, thiamine reduced apoptosis by approximately 50%. Thiamine also exhibits a cytoprotective effect by inhibiting apoptosis in the presence of hypoxia and by increasing the expression of heat shock protein HSP70. In a dose-dependent manner, thiamine inhibited the activity of effector caspase-3 and DNA fragmentation, which consequently reduced the percentage of cells undergoing apoptosis [38].

Thiamine diphosphate is essential for the activation of all transketolases [39]. In turn, transketolase plays a key role in the non-oxidative arm of the pentose–phosphate pathway (PPP), whose metabolites are able to feed its oxidative arm, which in turn supplies the body’s cells with NADPH. NADPH is used to maintain reduced glutathione (GSH) and thioredoxin, which are the main antioxidants in cells [40]. A study published in 2016 by Xu and her team showed that transketolase inhibition causes a decrease in NADPH levels, which impairs the cell’s ability to neutralize ROS [41]. Interestingly, this mechanism may be used to sensitize liver cancer cells to the drug sorafenib, and the mechanism is based precisely on the impairment of the ability to defend against drug-related oxidative stress.

## 4. Oxidative Stress Reduction

Thiamine has antioxidant properties that help reduce oxidative stress in cells. In thiamine deficiency, inflammatory processes and production of reactive oxygen species (ROS) increase, and oxidative stress intensifies [42]. Oxidative stress can cause DNA damage, meaning that, by mitigating this stress, thiamine indirectly supports the maintenance of DNA integrity. Interestingly, bacteria that endogenously produce thiamine use it as a regulator of oxidative stress and ROS. During oxidative stress, when repair enzymes of the mismatch repair (MMR) system are inhibited, the bacterial cell induces thiamine production, which serves as an ROS scavenger [43]. DNA microarray analyses showed that thiamine biosynthesis genes were commonly stimulated in Thermus thermophilus strains lacking mutS, mutL, and mutS2 (these genes cooperate in repairing mutagenic oxidative DNA damage, such as 8OG and 5-formyluracil).

**Figure 2:**
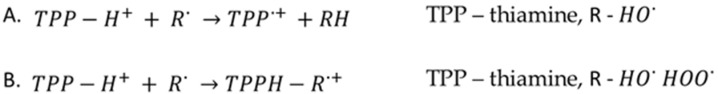
(**A**) The formal hydrogen transfer mechanism. (**B**) The radical adduct formation mechanism [45,48,49]. The FHT mechanism results in the neutralization of the aggressive radical by attaching hydrogen to it, thereby quenching it and forming a more stable radical derived from the antioxidant. In contrast, the RAF mechanism results in the formation of a radical adduct that is less reactive than the original radical.

Apart from playing the role of a cofactor for several enzymes, vitamin B1 may serve as a direct ROS scavenger [44]. Vitamin B1 is an exogenous antioxidant with the ability to scavenge the free radicals HO^●^ and HOO^●^ [45]. HO^●^ is an important ROS because it reacts with almost any organic biomolecule (e.g., DNA or proteins) at a diffusion-controlled rate [46]. In turn, HOO^●^, causing peroxidation of cardiolipin and other unsaturated lipids, damages, among other things, the mitochondria [47]. B1 removes HO^●^ based on the principles of the formal hydrogen transfer (FHT) and radical adduct formation (RAF) mechanisms; however, in the case of the HOO^●^ radical, only the RAF mechanism is effective (Figure 2) [48,49]. Direct antioxidant properties of thiamine are the highest among B vitamins, though they are still approximately three times weaker than in vitamin C [50]. However, it seems that the strong antioxidant and protective effects are primarily related to the indirect antioxidant effects of intracellular thiamine accumulation. The direct antioxidant effects of thiamine and thiamine diphosphate contribute to the overall protection to only a small extent [51]. In addition, thiamine protects hepatocytes from oxidative damage and iron-catalyzed oxidative stress by reducing lipid peroxidation, mitochondrial and protein damage, and DNA oxidation. In in vitro experiments assessing various compounds for their ability to shield isolated rat hepatocytes against the DNA damage caused by Fe-dependent oxidative stress, thiamine (1 mM) displayed a strong protective effect over time (1 h) when compared with cells with Fe-induced oxidative stress (*p* < 0.05). Additionally, thiamine demonstrated a significant capacity for binding iron, which helped prevent the formation of hydroxyl radicals by approximately 40%. Consequently, the action of thiamine protects cells and tissues from the harmful effects of oxidative stress [52].

Thiamine is a coenzyme, in addition to the previously mentioned transketolases, 2-Hydroxyacyl-CoAlase, pyruvate dehydrogenase, α-ketoglutarate dehydrogenase complex and α-branched ketoacid dehydrogenase, which are closely related to carbohydrate metabolism [53,54].

**Figure 3:**
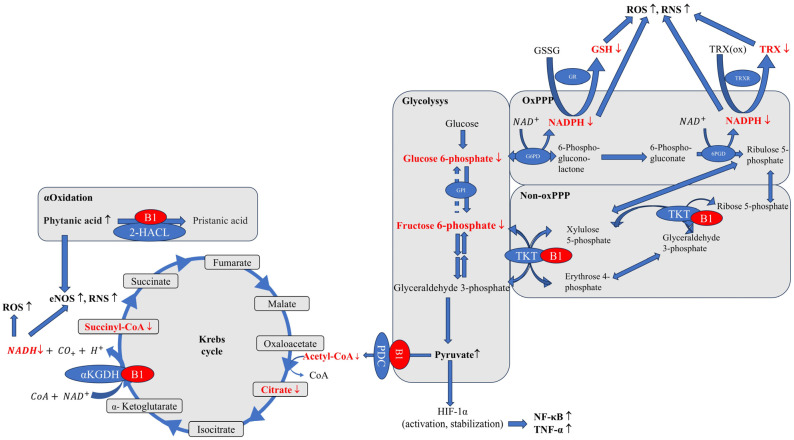
The influence of vitamin B1 on the level of oxidative stress (according to [30,40,54]). Thiamine deficiency inhibits PDC activity and contributes to the accumulation of pyruvate and the deficiency of acetyl-CoA. The latter is used to convert oxaloacetate to citrate in the Krebs cycle. Pyruvate accumulation causes activation and stabilization of HIF-1, thereby increasing NF-κB and TNF-α levels and the inflammatory response. Thiamine deficiency also inhibits TKT activity, which, by blocking the supply of non-oxPPP metabolites to ox-PPP, decreases NADPH levels. This NADPH is used to maintain normal GSH and TRX levels, and its deficiency impairs the ability of cells to neutralize ROS and RNS. The decrease in αKGDH activity caused by B1 deficiency limits NADH production, leading to increased ROS and activates eNOS, which increases RNS. Inhibition of 2-HALC activity in the α-oxidation process during vitamin B1 deficiency contributes to the accumulation of phytanic acid, which stimulates eNOS activity, while increasing RNS levels use glutathione reserves. ROS—reactive oxygen species, RNS—reactive nitrogen species, eNOS—endothelial nitric oxide synthase, 2-HACL—2-hydroxyacyl-CoA Lyase, NAD+/NADH—nicotinamide adenine dinucleotide, NADP+/NADPH—nicotinamide adenine dinucleotide phosphate, αKGDH—α-ketoglutarate dehydrogenase, CoA—coenzyme A, acetyl-CoA-acetyl coenzyme A, PDC—pyruvate dehydrogenase, HIF-1—hypoxia-induced factor-1α, NF-κB—nuclear factor kappa-B, TNF-α—tumor necrosis factor-α, GPI—glucose-6-phosphate isomerase, oxPPP—oxidative branch of the pentose–phosphate pathway, Non-oxPPP—non-oxidative branch of the pentose–phosphate pathway, G6PD—glucose-6-phosphate dehydrogenase, 6PGD—6-phosphogluconate dehydrogenase, GR—glutathione reductase, GSH—glutathione, GSSG—glutathione disulfide, TRX—thioredoxin, TRX(ox)—oxidized thioredoxin, TRXR—thioredoxin reductase, TKT—transketolase; Red color—chemical compounds whose deficiency occurs with vitamin B1 deficiency.

Thiamine is an essential cofactor of enzymes crucial to the Krebs cycle and ATP production. Its deficiency inhibits pyruvate dehydrogenase (PDC), leading to pyruvate accumulation and a metabolic shift to lactate fermentation, resulting in lactate acidosis and a decreased ATP level [53] (Figure 3).

The nervous system is the most sensitive to the toxic effects of pyruvic acid. In vivo and in vitro cellular studies suggest that thiamine deficiency increases the production of reactive oxygen species (ROS), generating oxidative stress in the brain and neuronal tissues [55,56,57]. This may be due to several factors, including mitochondrial dysfunction. Mitochondria are a key source of ROS and a target for thiamine deficiency. The availability of thiamine as a cofactor for enzymes acting at entry points and junctions for glucose, fatty acid, and amino acid pathways determines molecular oxygen homeostasis and ATP production in mitochondria. Thiamine deficiencies make mitochondria unable to use oxygen effectively even if it is supplied in adequate amounts. Such a condition is known as pseudohypoxia [58]. Insufficient ATP production disrupts oxidative phosphorylation, initiating a series of adverse effects: increased vascular reactivity, inflammation, and cell apoptosis. If the above processes are severe or chronic, they ultimately lead to organ dysfunction or failure [59].

At the same time, pyruvate accumulation stabilizes and activates hypoxia-induced factor-1α (HIF-1α) (even in the body’s physiological oxygenated state) which increases oxidative stress and induces an inflammatory response in the form of, for example, activation of nuclear factor kappa-B (NF-κB) and increased expression of tumor necrosis factor-α (TNF-α) [60,61] (Figure 3). Thiamine deficiency increases amounts of pro-inflammatory cytokines and chemokines, and contributes to neuroinflammation. Neuroinflammation, in turn, may alter mitochondrial function and increase oxidative stress. Reduced glutathione (GSH) levels have been observed in mouse brains after 8 and 10 days of thiamine deficiency compared with a control group [62]. Studies have shown a significant decrease in the activity of antioxidant enzymes, including catalase, superoxide dismutase, glutathione S-transferase, glutathione peroxidase, and glutathione reductase, along with an increase in lipid peroxidation in the brains of TD mice compared with a control group. Additionally, thiamine deficiency induces various changes in microglia, mast cells, endothelial cells, and astrocytes. All of these elements combine to cause neuronal death [63].

In this section, benfotiamine should be mentioned. It is a lipophilic thiamine derivative widely used in the treatment of vitamin B1 deficiency [42]. At the same time, the use of benfotiamine has a positive effect on oxidative stress, which has been studied in the context of neurodegenerative diseases, heart disease and kidney disease—mainly on an animal model. In neurodegenerative diseases like Alzheimer’s, benfotiamine activates the transcription factor Nrf2, leading to increased expression of genes encoding antioxidant enzymes such as heme oxygenase 1 (HO-1) and quinone reductase 1 (NQO1) [64]. This mechanism reduces oxidative stress and improves mitochondrial function. In addition, benfotiamine reduces the oxidative stress induced by ethanol consumption in an animal model of acute ethanol intoxication [65]. Treatment with thiamine or benfotiamine after a high dose of ethanol showed a protective effect against liver damage. The groups receiving thiamine or benfotiamine demonstrated correct values of liver damage markers, alanine aminotransferase (ALT), and aspartate aminotransferase (AST). Lipid peroxidation parameters were consistently lower in the groups receiving treatment with either form of thiamine. On the other hand, protein oxidation parameters were better in the case of benfotiamine treatment. Decades have passed since it was shown that alcohol and its metabolite, i.e., acetaldehyde, leads to irreversible protein damage, apoptotic cell death [66,67,68], and organ damage, including the brain [69]. Recent studies have shown that alcohol alters the brain biochemistry of chronically alcohol-abusing mice [70]. Alcohol exposure significantly increases brain GABA levels, decreases the expression of neurotrophic/growth factors, and increases the expression of neuroinflammatory markers such as interleukin-6 (IL-6), tumor necrosis factor-alpha (TNF-α), monocyte chemotactic protein-1 (MCP-1), and CC chemokine receptor 2 (CCR2). Alcohol also causes oxidative and endoplasmic reticulum (ER) stress, inducing neurodegeneration and gliosis via a proapoptotic cascade. Furthermore, alcohol inhibits the expression of thiamine transporters in the brain and decreases blood thiamine levels. Recent evidence suggests that the depletion of different vitamins due to alcohol consumption may also play a role in alcohol-induced organ and tissue damage [71,72,73]. In alcoholic cardiomyopathy, thiamine administration reverses ethanol-induced cytoplasmic damage, protecting against ethanol-induced changes in membrane fluidity and stability. Vitamin B1 abolishes acetaldehyde-induced protein damage and apoptotic cell death. Aberle et al. [67] suggest that vitamin B1-induced cardioprotection is related to the antagonism of oxidative stress in acetaldehyde-induced cell damage.

It also appears that the use of benfotiamine in oxidative stress control is more effective than the use of thiamine [42]. Above that, benfotiamine counteracts glucose-induced toxicity in both mouse models and diabetic dialysis patients [74]. Thiamine, as a coenzyme of transketolase, redirects glyceraldehyde-3-phosphate to the pentose–phosphate cycle and may prevent hyperglycemia-induced cell damage. It also shows beneficial effects in pathways associated with diabetes complications by acting through the same mechanisms as thiamine, i.e., controlling ROS generation through control of the PPP rather than directly capturing ROS [75].

Impaired α-ketoglutarate dehydrogenase (αKGDH) activity reduces NADH production, disrupting respiratory chain function and ATP synthesis. αKGDH is closely associated with the matrix side of the inner membrane [76,77]. It results in a significant impairment of energy metabolism, especially in tissues with high energy requirements, such as the brain [54]. The metabolic dysfunction that manifests most quickly in thiamine deficiency is the inhibition of αKGDH activity. Decreased αKGDH activity increases ROS production and up-regulates endothelial nitric oxide synthase (eNOS) activity, which also increases amounts of reactive nitrogen species (RNS) [78].

Fatty acid metabolism, necessary for supplying acetyl-CoA to the oxidative phosphorylation pathway, also relies on thiamine via B1-dependent 2-hydroxyacyl-CoA lyase, which is responsible for the alpha-oxidation of fatty acids, including phytanic acid [79]. Phytanic acid accumulation stimulates endothelial nitric oxide synthase (eNOS) activity, directly increasing reactive nitrogen species (RNS), and glutathione consumption, causing redox imbalance [80].

## 5. The Non-Coenzymatic Role of Thiamine

It is also worth mentioning the physiological contribution of non-coenzymatic functions of thiamine and its phosphate derivatives, including thiamine triphosphate (TTP), whose physiological role is still unclear [22]. Thiamine triphosphate concentrations are relatively high in erythrocytes, skeletal muscles, and neuronal cells [81]. In human erythrocytes, up to 10% of free thiamine diphosphate (TDP) can be converted to thiamine triphosphate, and this reaction is catalyzed (though very slowly) by adenylate kinase 1 (AK1) in accordance with the TDP + ADP reaction, resulting in TTP + AMP [82]. In the human brain, thiamine triphosphate levels are relatively high, constituting approximately 1% of total thiamine, and its synthesis is closely coupled to the respiratory chain and the participation of ATP synthase [83].

Thiamine triphosphate has been identified as a phosphate donor, phosphorylating 43K rapsyn, a peripheral protein specifically associated with the nicotinic acetylcholine receptor (nAChR) present in the postsynaptic membrane of the neuromuscular junction and the electrocyte [84]. Interestingly, thiamine triphosphate causes phosphorylation mainly of histidine residues. The presence of TTP in various tissues and organisms, ranging from bacteria and unicellular organisms to plants, animals, and humans, suggests that thiamine triphosphate plays a more general metabolic role than just being a donor of phosphate groups in the phosphorylation of synaptic proteins [75]. As the authors postulate, the process of protein phosphorylation by high-energy thiamine triphosphate may be part of a new, previously unknown signaling cascade in cells [85]. This may be confirmed by recent studies which show that thiamine and its derivatives participate in the modulation of protein expression through post-translational modifications. Pyridoxaldehyde kinase (PdxK) is associated with the circadian rhythm of physiological activity, which requires appropriate regulation of brain neurotransmitter metabolism. Interestingly, changes in the level of PdxK phosphorylation are dependent on thiamine and the time of day [86]. In studies, thiamine triphosphate was the best effector of human PdxK (hPdxK) and the only one among thiamine and its phosphates that significantly activated hPdxK at low ATP saturations, in the presence of Zn^2+^. Thiamine triphosphate also contributes to post-translational modification of other brain proteins. Circadian and thiamine-dependent changes in the allosteric regulation of brain glutamate dehydrogenase (GDH), resulting from the modulation of enzyme acetylation, have been shown [87]. Another study demonstrated the effect of thiamine on the activity of the pyruvate dehydrogenase complex (PDC), which is a key regulator of energy metabolism in the brain [88]. Thiamine treatment was shown to modulate the level of enzyme phosphorylation and its functions depending on the time of day, contributing to the regulation of the brain acetylation system and redox metabolism. In addition to functioning as coenzymes, thiamine and its derivatives modulate the activity of numerous protein targets. However, the consequences of this regulation remain insufficiently studied and confirmed, highlighting the need for further research in this area.

## 6. Discussion

A human’s health and well-being largely depend on the products they consume. Among these, some food products promote good health, while others are detrimental to it. The rising production and consumption of unhealthy foods represent a global crisis, with highly processed foods and beverages being major contributors to the growing rates of obesity. According to a 2015 report by the Pan American Health Organization (PAHO) of the World Health Organization, traditional diets based on unprocessed or minimally processed meals are increasingly being replaced by diets centered around ultra-processed food products and beverages [89].

Processed foods, including highly processed foods (HPF) and ultra-processed foods (UPF), often contain added thiamine, as well as large amounts of sugars and saturated fats. These ingredients can hinder energy metabolism and, despite the addition of thiamine, can lead to its intensive consumption. Recent research by Chang indicates that fortifying processed foods with vitamins and minerals has not effectively reduced the risk of cancer; in fact, it may promote and increase the risk of cancer [90]. Thiamine appears to have both cancer-promoting and anti-cancer properties, which vary depending on genomic and non-genomic factors [91]. High metabolic demands associated with neoplastic conditions may exacerbate thiamine deficiency. A recent prospective cohort study conducted at a single center confirmed significant thiamine deficiencies in oncology patients [92]. Could the reduction in peripheral thiamine/TPP be a consequence of intensive use by cancer cells? Interesting findings by Comín-Anduix et al. [93] reveal that a dose of thiamine 25 times higher than the recommended daily allowance (RDA) significantly stimulates tumor growth by 164% compared with a control group. However, at extremely high doses—approximately 2500 times the RDA—thiamine supplementation had the opposite effect; it inhibited tumor growth by 10%. Importantly, the authors conclude that moderate doses of thiamine to correct thiamine deficiencies common in oncology patients may have undesirable side effects, i.e., the promotion of tumor growth. Conversely, some studies indicate that thiamine intake is not associated with an increased risk of developing common cancers in women, including breast, endometrial, ovarian, colon, and lung cancers [94]. This highlights a need for further research on thiamine supplementation for nutritional support, especially considering its unknown oncogenic potential. Future studies should aim to differentiate the protective effects of vitamins in healthy individuals from their impact on cancer patients.

## 7. Conclusions

Viewing nutritional issues solely as concerns for populations with low economic status or food security overlooks other significant factors. It is crucial to consider the impact of food contamination, the choice of low-quality food (rather than necessity), and the effects of stress issues that also affect affluent populations. Failing to acknowledge these aspects can create a misleading sense of security. Furthermore, it is important to recognize that excessive food consumption is also a nutritional problem, as it can disrupt the body’s metabolic processes.

Consider thiamine, which is a vitamin associated with carbohydrate metabolism and energy production. Thiamine is particularly needed during times of increased metabolic activity. A state of increased exertion can deplete thiamine resources. Inflammation also contributes to the development of thiamine deficiencies. Acute inflammatory conditions like infections can be an example of factors which significantly accelerates the onset of thiamine deficiency [7]. Additionally, as noted by Mates et al., weight loss, loss of muscle or fat, and protein malnutrition are key indicators that may suggest the presence of thiamine deficiency. Moreover, an over-reliance on processed foods fortified with thiamine may obscure deficiencies in other essential nutrients and contribute to imbalanced dietary patterns. Nutritional needs should be provided primarily through good quality food and proper diet. Ongoing research and educational initiatives are crucial for enhancing food security and improving the overall quality of the population’s diet.

## Figures and Tables

**Table 1:** Table summarizing the key aspects of thiamine (vitamin B1), which combines thiamine’s properties, deficiency/excess implications, and guidance across special populations (according to [1,2,12,13,14,15,18]).

Aspect of Thiamine	Details & Guidance
Properties of Thiamine	Daily Requirements: provided primarily through diet, ~1.1 mg/day for women, ~1.2 mg/day for men; pregnant women have a thiamine intake of up to 1.4 mg/d
	Food Sources: Whole grains, legumes, lean meats
	Stability: Heat sensitive—cooking losses common
Deficiency	At-Risk Groups: Alcoholism, malabsorption, bariatric surgery, chronic diuretic use
	Related Diseases: Beriberi (wet: cardiovascular; dry: neurological) Wernicke–Korsakoff syndrome (in alcoholics), lactic acidosis, irritability, fatigue, poor memory
Excess	High-Dose Benefits: Investigated in hyperglycemic complications, post-stroke recovery
	Toxicity: The human body excretes excess thiamine in the urine. Toxicity resulting from high thiamine intake from food or supplements has not been established. There is no established upper limit of thiamine intake that causes toxicity
Specific Patient/Populations	Pregnancy/Lactation: Increased needs—especially in hyperemesis gravidarum
	Pediatrics: Essential during rapid growth—ensure adequate intake through diet or supplements in cases of mothers malnutrition or exclusive formula feeding Diabetes: Possible benefits in preventing microvascular complications; supplements should be considered (e.g., benfotiamine) Heart failure: Thiamine levels may be decreased in patients with heart failure Elderly: About 20% to 30% of elderly people are thiamine deficient. This may be due to inadequate intake of thiamine-rich foods, chronic health problems, and polypharmacy

## Data Availability

No new data were created or analyzed in this study. Data sharing is not applicable to this article.

## Abbreviations

The following abbreviations are used in this manuscript:

2-HACL	2-hydroxyacyl-CoA lyase
6PGD	6-phosphogluconate dehydrogenase
CoA	Coenzyme A, acetyl-CoA-acetyl coenzyme A
eNOS	Endothelial nitric oxide synthase
G6PD	Glucose-6-phosphate dehydrogenase
GPI	Glucose-6-phosphate isomerase
GR	Glutathione reductase
GSH	Glutathione
GSSG	Glutathione disulfide
HIF-1	Hypoxia-induced factor-1α
NAD+/NADH	Nicotinamide adenine dinucleotide
NADP+/NADPH	Nicotinamide adenine dinucleotide phosphate
NF-κB	Nuclear factor kappa-B
Non-oxPPP	Non-oxidative branch of pentose–phosphate pathway
oxPPP	Oxidative branch of pentose–phosphate pathway
PDC	Pyruvate dehydrogenase
PdxK	Pyridoxaldehyde kinase
PPP	Pentose–phosphate pathway
RNS	Reactive nitrogen species
ROS	Reactive oxygen species
TDP	Thiamine diphosphate, also known as thiamine pyrophosphate
TKT	Transketolase
TMP	Thiamine monophosphate
TNF-α	Tumor necrosis factor-α
TRX	Thioredoxin
TRX(ox)	Oxidized thioredoxin
TRXR	Thioredoxin reductase
TTP	Thiamine triphosphate
αKGDH	α-ketoglutarate dehydrogenase

## References

[1] Uchi T., Konno S., Kihara H., Sugimoto H. (2023). Thiamine deficiency unrelated to alcohol consumption presented with urinary retention and Wernicke’s encephalopathy: A case report. Clin. Case Rep..

[2] EFSA Panel on Dietetic Products, Nutrition and Allergies (NDA), Turck D., Bresson J., Burlingame B., Dean T., Fairweather-Tait S., Heinonen M., Hirsch-Ernst K.I., Mangelsdorf I., McArdle H.J. (2016). Dietary reference values for thiamin. EFSA J..

[3] Combs G.F. (2012). Thiamin. The Vitamins.

[4] Oudman E., Wijnia J.W., Oey M.J., van Dam M., Postma A. (2021). Wernicke-Korsakoff syndrome despite no alcohol abuse: A summary of systematic reports. J. Neurol. Sci..

[5] Schoenenberger A.W., Schoenenberger-Berzins R., der Maur C.A., Suter P.M., Vergopoulos A., Erne P. (2012). Thiamine supplementation in symptomatic chronic heart failure: A randomized, double-blind, placebo-controlled, cross-over pilot study. Clin. Res. Cardiol..

[6] Keith M.E., Walsh N.A., Darling P.B., Hanninen S.A., Thirugnanam S., Leong-Poi H., Sole M.J. (2009). B-Vitamin Deficiency in Hospitalized Patients with Heart Failure. J. Am. Diet. Assoc..

[7] Mates E., Alluri D., Artis T., Riddle M.S. (2021). A Retrospective Case Series of Thiamine Deficiency in Non-Alcoholic Hospitalized Veterans: An Important Cause of Delirium and Falling?. J. Clin. Med..

[8] Rakotoambinina B., Hiffler L., Gomes F. (2021). Pediatric thiamine deficiency disorders in high-income countries between 2000 and 2020: A clinical reappraisal. Ann. N. Y. Acad. Sci..

[9] Kim Y.N., Cho Y.O. (2019). Prevalent Low Thiamine Status Among Adults Living in Seoul Metropolitan Area (South Korea). Int. J. Vitam. Nutr. Res..

[10] Institute of Medicine (US) Standing Committee on the Scientific Evaluation of Dietary Reference Intakes and its Panel on Folate, Other B Vitamins, and Choline (1998). Thiamin. Dietary Reference Intakes for Thiamin, Riboflavin, Niacin, Vitamin B6, Folate, Vitamin B12, Pantothenic Acid, Biotin, and Choline.

[11] Elmadfa I., Majchrzak D., Rust P., Genser D. (2001). The thiamine status of adult humans depends on carbohydrate intake. Int. J. Vitam. Nutr. Res..

[12] Duc H.N., Oh H., Yoon I.M., Kim M.S. (2021). Association between levels of thiamine intake, diabetes, cardiovascular diseases and depression in Korea: A national cross-sectional study. J. Nutr. Sci..

[13] Alaei-Shahmiri F., Soares M.J., Zhao Y., Sherriff J. (2015). The impact of thiamine supplementation on blood pressure, serum lipids and C-reactive protein in individuals with hyperglycemia: A randomised, double-blind cross-over trial. Diabetes Metab. Syndr..

[14] Alaei Shahmiri F., Soares M.J., Zhao Y., Sherriff J. (2013). High-dose thiamine supplementation improves glucose tolerance in hyperglycemic individuals: A randomized, double-blind cross-over trial. Eur. J. Nutr..

[15] Ardhi M.S., Hamdan M., Romdhoni A.C. (2023). Effect of Thiamine on Serum Glutamate in Ischemic Stroke Animal Model. Pharmacogn. J..

[16] Oudman E. (2020). Wernicke encephalopathy in patients with depression: A systematic review. Psychiatry Clin. Neurosci..

[17] Zhang G., Ding H., Chen H., Ye X., Li H., Lin X., Ke Z. (2013). Thiamine nutritional status and depressive symptoms are inversely associated among older Chinese adults. J. Nutr..

[18] Martel J.L., Kerndt C.C., Doshi H., Sina R.E., Franklin D.S. (2025). Vitamin B1 (Thiamine). StatPearls [Internet].

[19] National Center for Biotechnology Information PubChem Compound Summary for CID 6042, Vitamin B1. https://pubchem.ncbi.nlm.nih.gov/compound/vitamin-B1.

[20] National Center for Biotechnology Information PubChem Compound Summary for CID 1132, Thiamine-Pyrophosphate. https://pubchem.ncbi.nlm.nih.gov/compound/Thiamine-pyrophosphate.

[21] National Center for Biotechnology Information PubChem Compound Summary for CID 511, Thiamine Triphosphate. https://pubchem.ncbi.nlm.nih.gov/compound/Thiamine-Triphosphate.

[22] Bettendorff L., Wins P. (2020). Biochemistry of Thiamine and Thiamine Phosphate Compounds. Encyclopedia of Biological Chemistry III.

[23] Patra K.C., Hay N. (2014). The pentose phosphate pathway and cancer. Trends Biochem. Sci..

[24] Xiao W., Wang R.S., Handy D.E., Loscalzo J. (2018). NAD(H) and NADP(H) Redox Couples and Cellular Energy Metabolism. Antioxid. Redox Signal..

[25] Ge T., Yang J., Zhou S., Wang Y., Li Y., Tong X. (2020). The Role of the Pentose Phosphate Pathway in Diabetes and Cancer. Front. Endocrinol..

[26] Pekovich S., Martin P., Singleton K. (1998). Thiamine Deficiency Decreases Steady-State Transketolase Pyruvate Dehydrogenase but not α-Ketoglutarate Dehydrogenase mRNA Levels in Three Human Cell Types. J. Nutr..

[27] Pekovich S.R., Martin P.R., Singleton C.K. (1996). Thiamine pyrophosphate–requiring enzymes are altered during pyrithiamine-induced thiamine deficiency in cultured human lymphoblasts. J. Nutr..

[28] Hostetler K.Y., Landau B.R. (1967). Estimation of the Pentose Cycle Contribution to Glucose Metabolism in Tissue in Vivo. Biochemistry.

[29] Kloska S.M., Pałczyński K., Marciniak T., Talaśka T., Miller M., Wysocki B.J., Davis P., Wysocki T.A. (2022). Queueing theory model of pentose phosphate pathway. Sci. Rep..

[30] TeSlaa T., Ralser M., Fan J., Rabinowitz J.D. (2023). The pentose phosphate pathway in health and disease. Nat. Metab..

[31] Cosentino C., Grieco D., Costanzo V. (2011). ATM activates the pentose phosphate pathway promoting anti-oxidant defence and DNA repair. EMBO J..

[32] Efferth T., Fabry U., Osieka R. (2016). DNA damage and apoptosis in mononuclear cells from glucose-6-phosphate dehydrogenase-deficient patients (G6PD Aachen variant) after UV irradiation. J. Leukoc. Biol..

[33] Riganti C., Gazzano E., Polimeni M., Aldieri E., Ghigo D. (2012). The pentose phosphate pathway: An antioxidant defense and a crossroad in tumor cell fate. Free Radical Biol. Med..

[34] Milanese C., Mastroberardino P.G. (2020). A perspective on DNA damage-induced potentiation of the pentose phosphate shunt and reductive stress in chemoresistance. Mol. Cell Oncol..

[35] Jiang P., Du W., Wang X., Mancuso A., Gao X., Wu M., Yang X. (2011). p53 regulates biosynthesis through direct inactivation of glucose-6-phosphate dehydrogenase. Nat. Cell Biol..

[36] Deshpande G.P., Patterton H.G., Faadiel Essop M. (2019). The human transketolase-like proteins TKTL1 and TKTL2 are bona fide transketolases. BMC Struct. Biol..

[37] McLure K.G., Takagi M., Kastan M.B. (2004). NAD+ modulates p53 DNA binding specificity and function. Mol. Cell Biol..

[38] Shin B.H., Choi S.H., Cho E.Y., Shin M.J., Hwang K.C., Cho H.K., Chung J.H., Jang Y. (2004). Thiamine attenuates hypoxia-induced cell death in cultured neonatal rat cardiomyocytes. Mol. Cells..

[39] Nauton L., Hecquet L., Théry V. (2021). QM/MM Study of Human Transketolase: Thiamine Diphosphate Activation Mechanism and Complete Catalytic Cycle. J. Chem. Inf. Model..

[40] Ju H.Q., Lin J.F., Tian T., Xie D., Xu R.H. (2020). NADPH homeostasis in cancer: Functions, mechanisms and therapeutic implications. Signal Transduct. Target. Ther..

[41] Xu I.M., Lai R.K., Lin S.H., Tse A.P., Chiu D.K., Koh H.Y., Law C.T., Wong C.M., Cai Z., Wong C.C. (2016). Transketolase counteracts oxidative stress to drive cancer development. Proc. Natl. Acad. Sci. USA.

[42] Bozic I., Lavrnja I. (2023). Thiamine and benfotiamine: Focus on their therapeutic potential. Heliyon.

[43] Fukui K., Wakamatsu T., Agari Y., Masui R., Kuramitsu S. (2011). Inactivation of the DNA repair genes mutS, mutL or the anti-recombination gene mutS2 leads to activation of vitamin B1 biosynthesis genes. PLoS ONE.

[44] Okai Y., Higashi-Okai K., FSato E., Konaka R., Inoue M. (2007). Potent radical-scavenging activities of thiamin and thiamin diphosphate. J. Clin. Biochem. Nutr..

[45] Nga N.T.T., Quang D.D. (2019). Unraveling the antioxidant potential of thiamine: Thermochemical and kinetics studies in aqueous phase using DFT. Viet J. Chem..

[46] Halliwell B., Adhikary A., Dingfelder M., Dizdaroglu M. (2021). Hydroxyl radical is a significant player in oxidative DNA damage in vivo. Chem. Soc. Rev..

[47] Panov A.V., Dikalov S.I. (2020). Cardiolipin, Perhydroxyl Radicals, and Lipid Peroxidation in Mitochondrial Dysfunctions and Aging. Oxid. Med. Cell Longev..

[48] Galano A., Raúl Alvarez-Idaboy J. (2019). Computational strategies for predicting free radical scavengers’ protection against oxidative stress: Where are we and what might follow?. Int. J. Quantum Chem..

[49] Carreon-Gonzalez M., Vivier-Bunge A., Alvarez-Idaboy J.R. (2019). Thiophenols Promising Scavengers of Peroxyl Radicals: Mechanisms kinetics. J. Comput. Chem..

[50] Gliszczynska-Swiglo A. (2006). Antioxidant activity of water soluble vitamins in the TEAC (trolox equivalent antioxidant capacity) and the FRAP (ferric reducing antioxidant power) assays. Food Chem..

[51] Sambon M., Napp A., Demelenne A., Vignisse J., Wins P., Fillet M., Bettendorff L. (2019). Thiamine and benfotiamine protect neuroblastoma cells against paraquat and β-amyloid toxicity by a coenzyme-independent mechanism. Heliyon.

[52] Mehta R., Dedina L., O’Brien P.J. (2011). Rescuing hepatocytes from iron-catalyzed oxidative stress using vitamins B1 and B6. Toxicol. Vitr..

[53] Sarandol E., Tas S., Serdar Z., Dirican M. (2020). Effects of thiamine treatment on oxidative stress in experimental diabetes. Bratisl. Med. J..

[54] Dhir S., Tarasenko M., Napoli E., Giulivi C. (2019). Neurological, Psychiatric, and Biochemical Aspects of Thiamine Deficiency in Children and Adults. Front. Psychiatry.

[55] Zhang Q., Yang G., Li W., Fan Z., Sun A., Luo J., Ke Z.J. (2011). Thiamine deficiency increases β-secretase activity and accumulation of β-amyloid peptides. Neurobiol. Aging.

[56] Yang G., Meng Y., Li W., Yong Y., Fan Z., Ding H., Wei Y., Luo J., Ke Z.J. (2011). Neuronal MCP-1 mediates microglia recruitment and neurodegeneration induced by the mild impairment of oxidative metabolism. Brain Pathol..

[57] Wang X., Xu M., Frank J.A., Ke Z.J., Luo J. (2017). Thiamine deficiency induces endoplasmic reticulum stress and oxidative stress in human neurons derived from induced pluripotent stem cells. Toxicol. Appl. Pharmacol..

[58] Sweet R.L., Zastre J.A. (2013). HIF1-α-mediated gene expression induced by vitamin B1 deficiency. Int. J. Vitam. Nutr. Res..

[59] Bhatti J.S., Bhatti G.K., Reddy P.H. (2017). Mitochondrial dysfunction and oxidative stress in metabolic disorders—A step towards mitochondria based therapeutic strategies. Biochim. Biophys. Acta Mol. Basis Dis..

[60] Zhao M., Wang S., Zuo A., Zhang J., Wen W., Jiang W., Chen H., Liang D., Sun J., Wang M. (2021). HIF-1α/JMJD1A signaling regulates inflammation and oxidative stress following hyperglycemia and hypoxia-induced vascular cell injury. Cell Mol. Biol. Lett..

[61] Zera K., Zastre J. (2018). Stabilization of the hypoxia-inducible transcription Factor-1 alpha (HIF-1α) in thiamine deficiency is mediated by pyruvate accumulation. Toxicol. Appl. Pharmacol..

[62] Sharma A., Bist R., Bubber P. (2013). Thiamine deficiency induces oxidative stress in brain mitochondria of Mus musculus. J. Physiol. Biochem..

[63] Chauhan A., Srivastva N., Bubber P. (2017). Thiamine Deficiency Induced Dietary Disparity Promotes Oxidative Stress and Neurodegeneration. Indian J. Clin. Biochem..

[64] Tapias V., Jainuddin S., Ahuja M., Stack C., Elipenahli C., Vignisse J., Gerges M., Starkova N., Xu H., Starkov A.A. (2018). Benfotiamine treatment activates the Nrf2/ARE pathway and is neuroprotective in a transgenic mouse model of tauopathy. Hum. Mol. Genet..

[65] Portari G.V., Ovidio P.P., Deminice R., Jordão A.A. (2016). Protective effect of treatment with thiamine or benfotiamine on liver oxidative damage in rat model of acute ethanol intoxication. Life Sci..

[66] Hintz K.K., Relling D.P., Saari J.T., Borgerding A.J., Duan J., Ren B.H., Kato K., Epstein P.N., Ren J. (2003). Cardiac overexpression of alcohol dehydrogenase exacerbates cardiac contractile dysfunction, lipid peroxidation, and protein damage after chronic ethanol ingestion. Alcohol. Clin. Exp. Res..

[67] Aberle N.S., Burd L., Zhao B.H., Ren J. (2004). Acetaldehyde-induced cardiac contractile dysfunction may be alleviated by vitamin B1 but not by vitamins B6 or B12. Alcohol Alcohol..

[68] Duan J., McFadden G.E., Borgerding A.J., Norby F.L., Ren B.H., Ye G., Epstein P.N., Ren J. (2002). Overexpression of alcohol dehydrogenase exacerbates ethanol-induced contractile defect in cardiac myocytes. Am. J. Physiol. Heart Circ. Physiol..

[69] Zahr N.M., Pfefferbaum A. (2017). Alcohol’s Effects on the Brain: Neuroimaging Results in Humans and Animal Models. Alcohol. Res..

[70] Xu H., Li H., Liu D., Wen W., Xu M., Frank J.A., Chen J., Zhu H., Grahame N.J., Luo J. (2021). Chronic Voluntary Alcohol Drinking Causes Anxiety-like Behavior, Thiamine Deficiency, and Brain Damage of Female Crossed High Alcohol Preferring Mice. Front. Pharmacol..

[71] Marik P.E., Liggett A. (2019). Adding an orange to the banana bag: Vitamin C deficiency is common in alcohol use disorders. Crit. Care.

[72] Nicoll F., Gerasimidis K., Forrest E. (2022). The Role of Micronutrients in the Pathogenesis of Alcohol-Related Liver Disease. Alcohol Alcohol..

[73] Ferdouse A., Agrawal R.R., Gao M.A., Jiang H., Blaner W.S., Clugston R.D. (2022). Alcohol induced hepatic retinoid depletion is associated with the induction of multiple retinoid catabolizing cytochrome P450 enzymes. PLoS ONE.

[74] Schupp N., Dette E.M., Schmid U., Bahner U., Winkler M., Heidland A., Stopper H. (2008). Benfotiamine reduces genomic damage in peripheral lymphocytes of hemodialysis patients. Naunyn-Schmied. Arch. Pharmacol..

[75] Forman H.J., Zhang H. (2021). Targeting oxidative stress in disease: Promise and limitations of antioxidant therapy. Nat. Rev. Drug Discov..

[76] Starkov A.A. (2013). An update on the role of mitochondrial α-ketoglutarate dehydrogenase in oxidative stress. Mol. Cell Neurosci..

[77] Starkov A.A., Fiskum G., Chinopoulos C., Lorenzo B.J., Browne S.E., Patel M.S., Beal M.F. (2004). Mitochondrial alpha-ketoglutarate dehydrogenase complex generates reactive oxygen species. J. Neurosci..

[78] Kruse M., Navarro D., Desjardins P., Butterworth R.F. (2004). Increased brain endothelial nitric oxide synthase expression in thiamine deficiency: Relationship to selective vulnerability. Neurochem. Int..

[79] Mezzar S., De Schryver E., Asselberghs S., Meyhi E., Morvay P.L., Baes M., Van Veldhoven P.P. (2017). Phytol-induced pathology in 2-hydroxyacyl-CoA lyase (HACL1) deficient mice. Evidence for a second non-HACL1-related lyase. Biochim. Biophys. Acta Mol. Cell Biol. Lipids.

[80] Borges C.G., Canani C.R., Fernandes C.G., Zanatta Â., Seminotti B., Ribeiro C.A., Leipnitz G., Vargas C.R., Wajner M. (2015). Reactive nitrogen species mediate oxidative stress and astrogliosis provoked by in vivo administration of phytanic acid in cerebellum of adolescent rats: A potential contributing pathomechanism of cerebellar injury in peroxisomal disorders. Neuroscience.

[81] Gangolf M., Czerniecki J., Radermecker M., Detry O., Nisolle M., Jouan C., Martin D., Chantraine F., Lakaye B., Wins P. (2010). Thiamine status in humans and content of phosphorylated thiamine derivatives in biopsies and cultured cells. PLoS ONE.

[82] Egi Y., Koyama S., Shioda T., Yamada K., Kawasaki T. (1992). Identification, purification and reconstitution of thiamin metabolizing enzymes in human red blood cells. Biochim. Biophys. Acta.

[83] Bettendorff L. (2021). Update on Thiamine Triphosphorylated Derivatives and Metabolizing Enzymatic Complexes. Biomolecules.

[84] Nghiem H.O., Bettendorff L., Changeux J.P. (2000). Specific phosphorylation of Torpedo 43K rapsyn by endogenous kinase(s) with thiamine triphosphate as the phosphate donor. FASEB J..

[85] Bettendorff L., Wins P. (2009). Thiamin diphosphate in biological chemistry: New aspects of thiamin metabolism especially triphosphate derivatives action other than as cofactors. FASEB J..

[86] Bunik V., Aleshin V., Nogues I., Kähne T., Parroni A., Contestabile R., Salvo M.L., Graf A., Tramonti A. (2022). Thiamine-dependent regulation of mammalian brain pyridoxal kinase in vitro and in vivo. J. Neurochem..

[87] Aleshin V.A., Mkrtchyan G.V., Kaehne T., Graf A.V., Maslova M.V., Bunik V.I. (2020). Diurnal regulation of the function of the rat brain glutamate dehydrogenase by acetylation and its dependence on thiamine administration. J. Neurochem..

[88] Aleshin V.A., Artiukhov A.V., Kaehne T., Graf A.V., Bunik V.I. (2021). Daytime Dependence of the Activity of the Rat Brain Pyruvate Dehydrogenase Corresponds to the Mitochondrial Sirtuin 3 Level and Acetylation of Brain Proteins, All Regulated by Thiamine Administration Decreasing Phosphorylation of PDHA Ser293. Int. J. Mol. Sci..

[89] Monteiro C.A., Cannon G., Moubarac J.C., Levy R.B., Louzada M.L.C., Jaime P.C. (2018). The UN Decade of Nutrition, the NOVA food classification and the trouble with ultra-processing. Public Health Nutr..

[90] Chang K., Gunter M.J., Rauber F., Levy R.B., Huybrechts I., Kliemann N., Millett C., Vamos E.P. (2023). Ultra-processed food consumption, cancer risk and cancer mortality: A large-scale prospective analysis within the UK Biobank. EClinicalMedicine.

[91] Niu C., Qiu W., Li X., Li H., Zhou J., Zhu H. (2022). Transketolase Serves as a Biomarker for Poor Prognosis in Human Lung Adenocarcinoma. J. Cancer.

[92] Boopathy D., Grahf D., Ross J., Hawatian K., Rammal J.A., Alaimo K., Miller J.B. (2025). Thiamine Deficiency Is Common and Underrecognized in Emergency Department Oncology Patients. J. Clin. Med..

[93] Comín-Anduix B., Boren J., Martinez S., Moro C., Centelles J.J., Trebukhina R., Petushok N., Lee W.N., Boros L.G., Cascante M. (2001). The effect of thiamine supplementation on tumour proliferation. A metabolic control analysis study. Eur. J. Biochem..

[94] Kabat G.C., Miller A.B., Jain M., Rohan T.E. (2008). Dietary intake of selected B vitamins in relation to risk of major cancers in women. Br. J. Cancer.

